# A modified Jarnagin-Blumgart classification better predicts survival for resectable hilar cholangiocarcinoma

**DOI:** 10.1186/s12957-015-0526-5

**Published:** 2015-03-11

**Authors:** Guoping Ding, Yifei Yang, Liping Cao, Wenchao Chen, Zhengrong Wu, Guixing Jiang

**Affiliations:** Department of Surgery, Second Affiliated Hospital, School of Medicine, Zhejiang University, Hangzhou, 310009 China

**Keywords:** Hilar cholangiocarcinoma, Stage, Survival, Jarnagin-Blumgart classification

## Abstract

**Background:**

Prediction of postoperative survival for hilar cholangiocarcinoma (HCCA) remains difficult although there have been a variety of clinical classification and staging systems. This study was designed to validate and compare some of the major HCCA staging systems in use today. In addition, we sought to build up a new staging system modified from Jarnagin-Blumgart (J-B) classification for HCCA, to predict survival better.

**Methods:**

A total of 154 consecutive cases of HCCA including 95 surgical patients between 2005 and 2014 were enrolled in this study. The clinical and pathological data were recorded retrospectively and three commonly used classification methods: Bismuth-Corlette (B-C) classification, TNM staging, and J-B classification were performed to analyze the correlations with resectability and survival. Chi-square test, Kaplan-Meier analysis, and kappa statistics were used to compare and confirm the relationships between the variables and survival.

**Results:**

For all 154 patients, the resection rate of J-B T1 was 68.6% (48/70), higher than that of J-B T2 (44.8%, *P* = 0.007). J-B T2 also showed a higher resectability than J-B T3 (19.2%, *P* = 0.025). There was no significant difference in resectability within the groups B-C type and TNM stages. We set up a new staging system based on J-B classification, tumor differentiation, distant metastasis (N2 or M1 of TNM stage), and resection integrality. The total survival predictive accuracy was 69.5% (kappa = 0.547), higher than that of TNM staging and J-B classification.

**Conclusions:**

J-B classification was more useful than B-C classification, while its value for predicting survival did not exceed TNM staging system. The new staging system, based on J-B classification, provides a better method to stratify HCCA patients during the operation.

## Background

Hilar cholangiocarcinoma (HCCA), which occurs at or near the junction of the left and right hepatic ducts, still remains a difficult clinical problem for surgeons because of its low resectability and poor prognosis [1]. Radical resection is the only potentially curative therapy for HCCA. Most patients with untreated HCCA have a median survival of 6 months [2]. Even for patients who underwent radical resections, the 5-year survival rate is only about 20% to 40% [3,4]. In recent years, surgical treatment of HCCA was more common than ever and the resection extent gradually expanded [5], while the staging and classification methods for HCCA fallen behind the surgical development. It still remains urgent to better stratify HCCA patients, paying more attention to long-term survival and low morbidity after operation.

At present, there exist a variety of clinical classification and staging systems for HCCA, including Bismuth-Corlette (B-C) classification [6], TNM staging of AJCC [7] and Jarnagin-Blumgart (J-B) classification [8], and so on. However, their criteria varied greatly and the prognostic accuracy has been questioned [9,10]. The mostly used B-C classification, which defines the longitudinal tumor spread along the bile duct, is inadequate for preoperative staging and predicting resectability. TNM staging system, which is based largely on pathological criteria, is a postoperative staging system and has little applicability for predicting resectability. As a preoperative clinical staging system, J-B classification is based on the factors related to local tumor extent within the hepatic portal and stratifies patients using noninvasive preoperative imaging. In a cohort of 380 HCCA patients at Memorial Sloan-Kettering Cancer Center (MSKCC), J-B classification predicted the resectability with a convincing accuracy and the resectability significantly decreased with increasing clinical T stage. Unsatisfactorily, this staging system was less useful for predicting survival [11].

It is clearly understandable and predictable that the prognosis of unresectable HCCA is extremely poor, while there was no practical staging system which was specially designed for potential resectable HCCA. This study was designed to validate and compare some of the major HCCA staging systems in use today, thus to evaluate their clinical value for predicting resectability and survival of HCCA after resection. In addition, we sought to build up a new staging system based on J-B classification and the risk factors associated with survival, which will compensate the defect of J-B classification in predicting survival.

## Patients and methods

### Ethics statement

The research protocol was reviewed and approved by the Research Ethics Committee of Second Affiliated Hospital, School of Medicine, Zhejiang University. All participants or their guardians gave written consent of their serum samples and medical information to be used for scientific research.

### Patients

From January 2005 to January 2014, a group of 154 consecutive HCCA patients, including 80 males (51.9%) and 74 females (48.1%) with a mean age of 62.3 years, were enrolled in the study. The patients were histologically confirmed of HCCA without previous or coexisting cancer.

There were 95 patients who underwent surgical treatments, including 56 males (58.9%) and 39 females (41.1%) with a mean age of 60.4 years (ranging from 29 to 84 years). Among the 95 HCCA patients, 61 cases (64.2%) underwent a R0 resection including 16 cases of left hepatectomy, 7 cases of right hepatectomy, 5 cases of quadrate hepatectomy, and 4 cases of left and caudate lobe resection (complete resection of the tumor, regional lymph nodes dissection); 18 patients (18.9%) underwent palliative resection (R1/R2, hepatic duct, and intestinal anastomosis); 3 patients (3.2%) underwent tumor biopsy and internal drainage (R2 resection); and 13 patients (13.7%) underwent surgical exploration and biopsy (R2 resection).

## Methods

Clinical and pathological data of patients were reviewed retrospectively and analyzed according to the NCCN Guidelines for hepatobiliary cancer [12]. The tumor location, portal vein involvement, and hepatic lobe atrophy were evaluated by preoperative radiographic examinations. Tumor invasion depth, lymph node (LN) involvement, and distant metastasis were determined by postoperative pathological examinations. B-C classification, TNM stage, and J-B classification were evaluated for each patient.

The survival time was defined as the time between the date of surgery and the date of either death or last contact. According to the survival time, patients were divided into the following three groups: poor prognosis, 12 months or less; moderate prognosis, 12 to 36 months; and good prognosis, above 3 years.

### Statistics analysis

All the clinical data were analyzed by the software of PASW statistics for windows (Version 18.0, SPSS Inc., Chicago, IL, USA). Numeration data were expressed as mean ± SD. Survival was analyzed by Kaplan-Meier analysis. Chi-square test was used to confirm the relationship between the variables and survival. The agreement between tumor stage and actual survival was analyzed by means of kappa statistics.

## Results

### Staging and surgical treatment

The distribution of all the patients by B-C classification, TNM stage, and J-B classification is shown in Table 1. The resection rates (radical resection and palliative resection) of B-C types I, II, III, and IV were 63.2% (24/38), 57.6% (19/33), 52.3% (34/65), and 11.1% (2/18), respectively (Table 1). There was no significant difference in resectability between B-C types I, II, and III (*P* = 0.558), although the B-C classification was correlated with surgical treatment. In this study, 59 patients (38.3%) who accepted nonsurgical treatment were unable for TNM staging. The resection rates were 100% (26/26), 93.8% (30/32), 84% (21/25), and 16.7% (2/12) for TNM stages I, II, III, and IV, respectively. There was also no significant difference between TNM stages I, II, and III (*P* = 0.085). For all 154 patients, the resection rate of J-B T1 was 68.6% (48/70), higher than that of J-B T2 (44.8%, *P* = 0.007). J-B T2 also showed a higher resectability than J-B T3 (19.2%, *P* = 0.025).

**Table 1:** **Analysis of staging method and surgical treatment**

	**Surgical treatment (** ***n*** **)**					***P***
	**Radical resection**	**Palliative resection**	**Internal drainage**	**Surgical exploration**	**Nonsurgical treatment**	
B-C classification (*n*)						0.000
I	19	5	0	2	12	
II	17	2	0	0	14	
III	24	10	2	3	26	
IV	1	1	1	8	7	
TNM stage (*n*)						0.000
I	26	0	0	0		
II	25	5	1	1		
III	9	12	0	4		
IV	1	1	2	8		
J-B classification (*n*)						0.000
T1	38	10	0	2	20	
T2	21	5	1	2	29	
T3	2	3	2	9	10	

Among the 95 HCCA patients, 61 cases underwent a R0 resection including 32 cases of combined hepatectomy. The other 29 cases underwent a major bile duct excision and lymphadenectomy with negative bile duct margins, including 19 cases of B-C type I and 10 cases of type II. For the 61 patients who underwent R0 resections, the mean removed LN count was 9.3 (ranging from 3 to 25) and there were 10 cases with LN metastasis. The total LN metastasis rate was 22.1% (21/95).

### Clinical factors associated with survival

**Figure 1:**
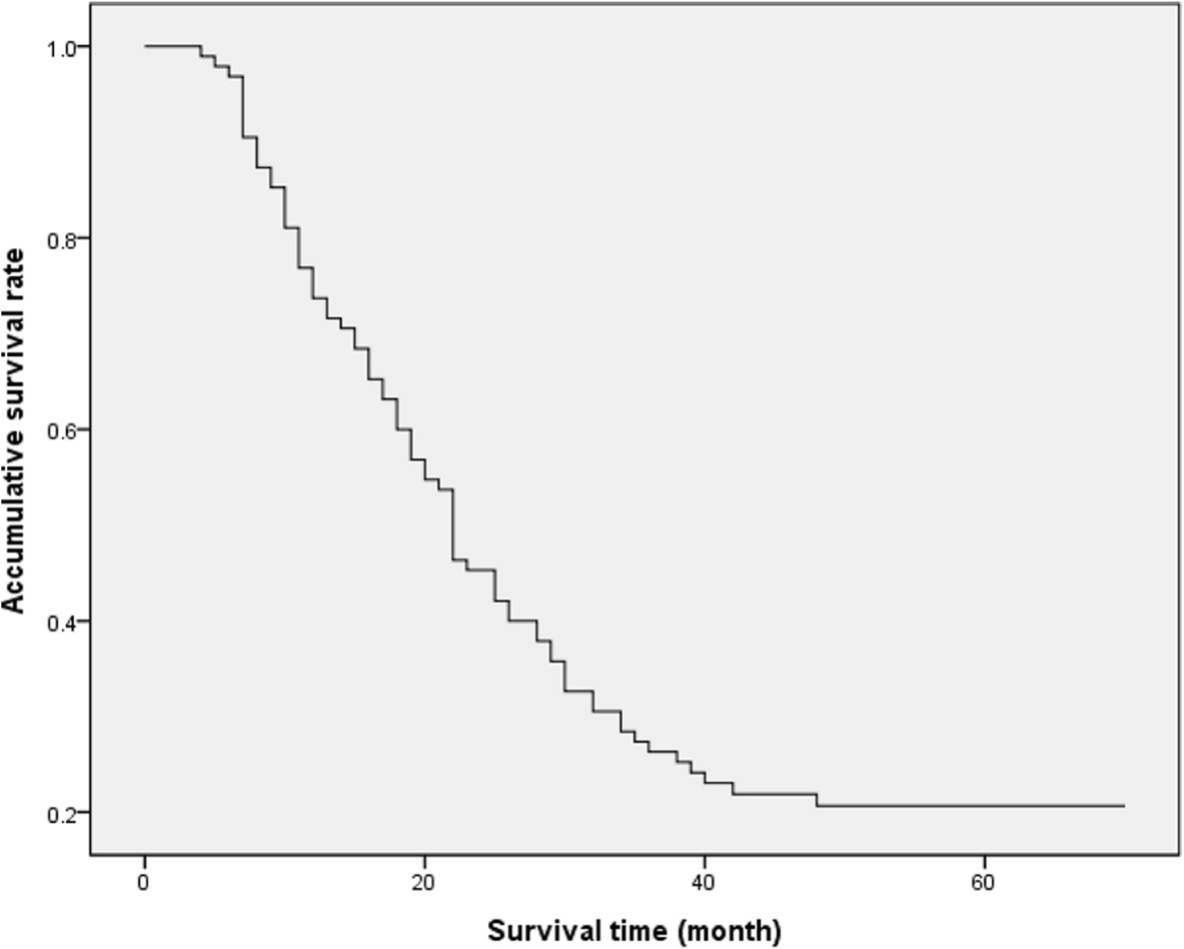
**Survival curve of 95 HCCA patients after surgical treatment.** The median survival time for all the 95 HCCA patients was 21.5 months. The 1-, 3-, and 5-year survival rates were 72%, 26%, and 21%, respectively.

To compare values of the three staging methods for predicting survival of HCCA, we analyzed the survival of 95 surgical patients. The main clinical and pathologic variables correlated with survival are shown in Table 2. The median survival time of was 21.5 months. The 1-, 3-, and 5-year accumulative survival rates were 72%, 26%, and 21%, respectively (Figure 1). The survival time of 75 patients who died ranged from 3 to 47 months, among which 27 (36%) died in the first year after resection, 43 (57.3%) died between 1 to 3 years, and 5 (6.7%) died between 3 to 5 years. Chi-square test indicated that TNM stage, J-B classification, tumor differentiation, LN involvement, distant metastasis, and margin status were significantly correlated with survival, while gender, age, upper abdominal pain, jaundice, and B-C classification were not (Table 1).

**Table 2:** **Analysis of variables correlated with overall survival of HCCA**

**Variables**	**Survival time (years)**				**Chi-square**	***P*** **value**
	**≤1**	**1 ~ 3**	**3 ~ 5**	**>5 or alive**		
Sex (*n*)					6.29	0.098
Male	13	25	0	11		
Female	14	18	5	9		
Ages (*n*, years)					7.37	0.599
≤40	0	0	0	1		
40 to 60	11	20	3	7		
60 to 80	15	23	2	12		
80 and above	1	0	0	0		
Upper abdominal pain (*n*)					4.19	0.242
No	10	24	4	10		
Yes	17	19	1	10		
Jaundice (*n*)					3.69	0.297
No	11	18	1	4		
Yes	16	25	4	16		
Cholangiolithiasis (*n*)					1.62	0.655
No	24	41	5	18		
Yes	3	2	0	2		
Tumor differentiation (*n*)					23.48	0.001
Well	10	19	4	14		
Moderate	5	19	1	6		
Poor	12	5	0	0		
Margin status (*n*)					50.91	0.000
R0 resections	3	33	5	20		
R1 resections	4	3	0	0		
R2 resections	20	7	0	0		
LN involvement (*n*)					26.19	0.000
N0	12	38	5	19		
N1	8	4	0	0		
N2	7	1	0	1		
Distant metastasis (*n*)					27.08	0.000
M0	16	42	5	20		
M1	11	1	0	0		
B-C classification (*n*)					13.02	0.162
I	4	14	1	9		
II	4	6	2	3		
III	6	13	2	3		
IV	13	10	0	5		
TNM staging (*n*)					47.27	0.000
I	2	11	1	12		
II	4	18	4	6		
III	10	13	0	2		
IV	11	1	0	0		
J-B classification (*n*)					33.79	0.000
T1	6	25	4	15		
T2	8	16	1	5		
T3	13	2	0	0

### The correlation of different classification methods and survival

**Figure 2:**
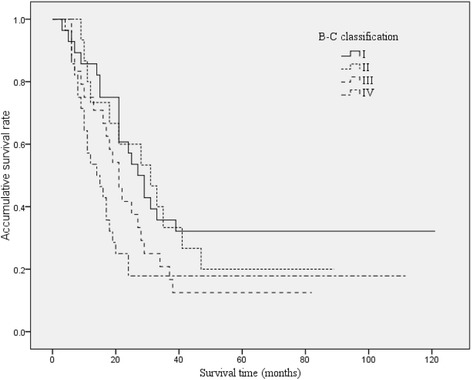
**Overall Kaplan-Meier survival stratified by Bismuth-Corlette classification for HCCA.** The median survival times were 27, 31, 21, and 14 months for B-C I, II, III, and IV types, respectively.

**Figure 3:**
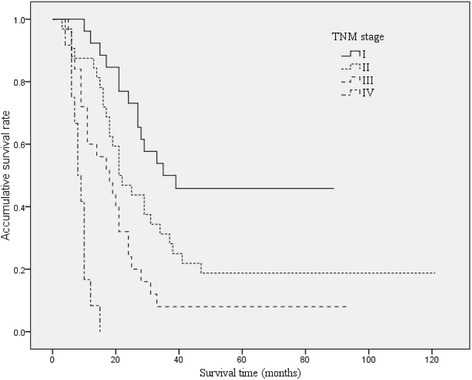
**Overall Kaplan-Meier survival stratified by TNM stage for HCCA.** The median survival times were 35, 21, 18, and 8 months for TNM I, II, III, IV stages, respectively.

**Figure 4:**
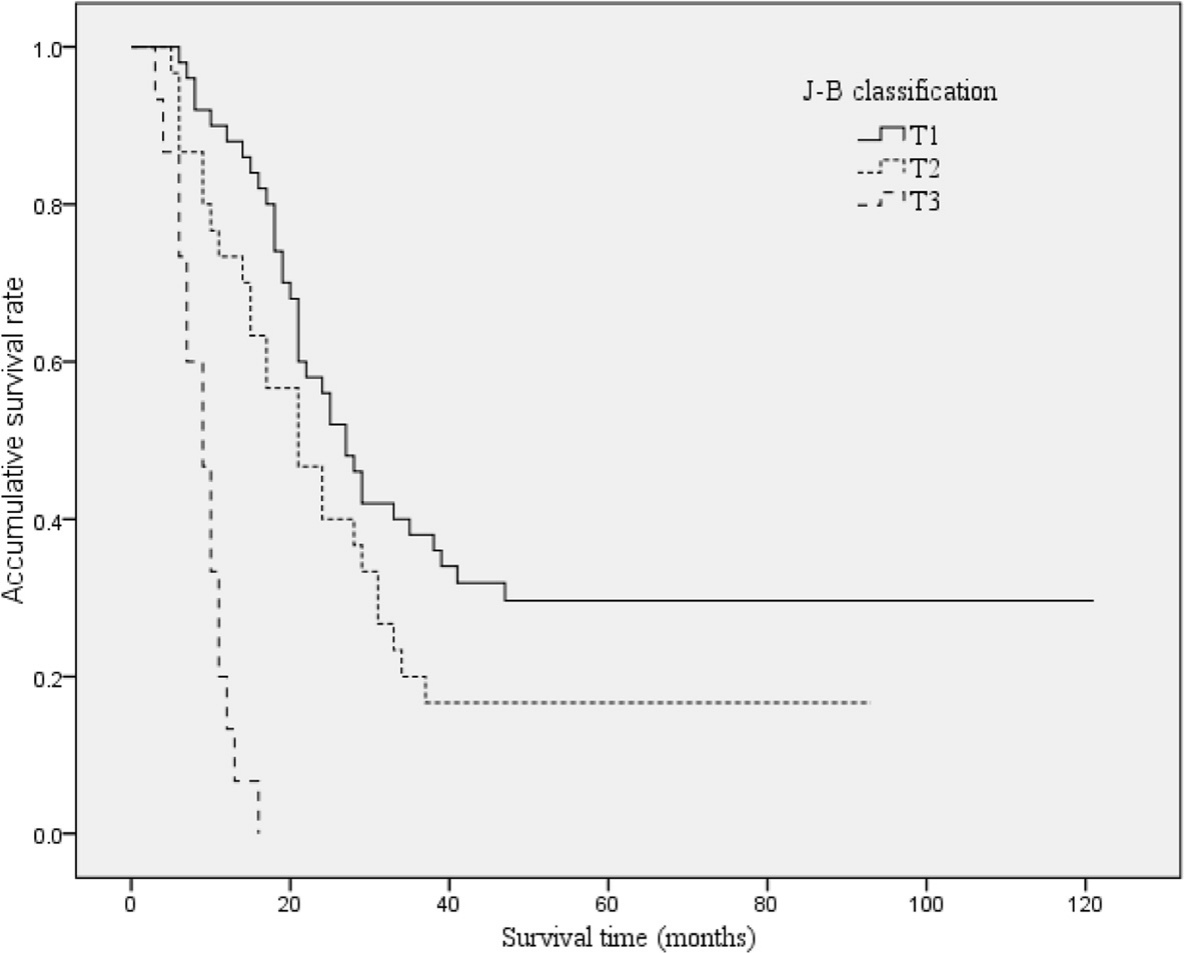
**Overall Kaplan-Meier survival stratified by Jarnagin-Blumgart classification for HCCA.** The median survival times were 27, 21, and 9 months for J-B T1, T2, and T3 stages, respectively.

The survival curves of HCCA patients with different B-C classification, TNM stage, and J-B classification were shown in Figures 2, 3, and 4. The median survival time were 27, 31, 21, and 14 months for B-C I, II, III, IV types; 35, 21, 18, and 8 months for TNM I, II, III, IV stages; and 27, 21, and 9 months for J-B T1, T2, and T3 stages, respectively.

The predicting accuracies of TNM stage I for good prognosis, stage II for moderate prognosis, and stages III and IV for poor prognosis were 50.0%, 56.3%, and 58% respectively (Table 3, *P* = 0.000, *Kappa* = 0.319). The total predictive accuracy of TNM stage for prognosis of HCCA was 54.2%. The predicting accuracies of J-B T1 for good prognosis, T2 for moderate prognosis, and T3 for poor prognosis were 38.0%, 53.3%, and 86.7%, respectively (Table 3, *P* = 0.000, kappa = 0.266). The total predictive accuracy of J-B classification was 50.5%.

**Table 3:** **Prediction of prognosis by the different staging system**

**Stage**	**Prognosis (** ***n*** **)**			**Accuracy (%)**
	**Good**	**Moderate**	**Poor**	
TNM stage				
I	13	11	2	50.0
II	10	18	4	56.3
III and IV	2	14	21	58
J-B classification				
T1	19	25	6	38.0
T2	6	16	8	53.3
T3	0	2	13	86.7
Modified J-B classification				
I	19	12	2	57.6
II	6	22	0	78.6
III	0	9	25	73.5

### The proposed staging system and survival

By the statistic analysis of risk factors correlated with survival, we set up a new intraoperative staging system based on J-B classification which has a considerable value for predicting HCCA resectability. The staging system is shown in Tables 4 and 5. The total score is calculated by adding the scores of preoperative J-B classification, intraoperative findings, and resection margin. The patients with total scores of 0, 1, and ≧2 are considered as stage I, stage II, and stage III, respectively.

**Table 4:** **The scoring criteria of the new staging system for hilar cholangiocarcinoma**

**Scores**	**Jarnagin-Blumgart classification**	**Intraoperative findings**	**Resection margin**
0	T1	High/moderate differentiation without distant metastasis	R0
1	T2	Poor differentiation	R1
2	T3	Distant metastasis	R2

**Table 5:** **The proposed staging system and significance for hilar cholangiocarcinoma**

**Total score**	**Stage**	**Prognosis**	**Predicting survival time (years)**
0	I	Well	>3
1	II	Moderate	1 to 3
2 to 6	III	Poor	≤1

**Figure 5:**
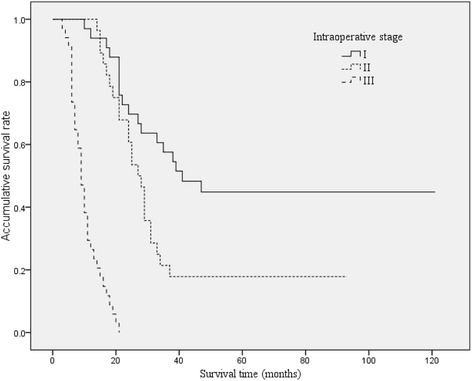
**Overall Kaplan-Meier survival stratified by the new staging system for HCCA.** By this staging system, the median survival times were 41, 27, and 9 months for stages I, II, and III, respectively.

By this staging system, the median survival times were 41, 27, and 9 months for stages I, II, and III, respectively (Figure 5). The predicting accuracies of stage I for good prognosis, stage II for moderate prognosis, and stage III for poor prognosis were 57.6%, 78.6%, and 73.5% respectively (Table 3, *P* = 0.000, kappa = 0.547). The total predictive accuracy was 69.5%, higher than that of TNM staging and J-B classification.

## Discussion

HCCA is the most common type of cholangiocarcinoma, comprising up to 50% to 70% of all bile duct carcinomas [12]. The incidence of HCCA has increased over the past two decades, and the overall survival of HCCA is still extremely poor [13,14]. Even after a radical resection, the 5-year survival rate rarely exceeds 40% [3,4,15]. A practical staging system, which can guide the optimal surgical treatment and predict postoperative survival with a convincing accuracy, is still urgently needed for surgeons [13]. In the present study, there were 61 cases (64.2%) that underwent a R0 resection in 95 patients. Major bile duct excision and lymphadenectomy with frozen section assessment of bile duct margins was performed for patients of B-C type I-II and without liver parenchyma involvement. Hilar bile duct resection with lymphadenectomy and combined hepatectomy was recommended for lesions of B-C type II-III or liver parenchyma involvement. The total LN metastasis rate was 22.1%, and the median survival time was 21.5 months, which were similar to the previous studies [16,17].

At present, there are a variety of clinical classification and staging methods for HCCA [6-8]. The B-C classification, which is commonly used for preoperative clinical classification of HCCA, does not include crucial information such as vascular involvement and distant metastases and therefore cannot identify nonresectable patients. Thus, it has been proved to have no correlation with resectability nor with survival [18]. The present study also showed that B-C classification had poor value for predicting resectability and survival. TNM staging system is the most common method for cancer staging, while its prognostic accuracy for HCCA has been questioned [9]. For HCCA, it seems inappropriate to define the T subgroups by transversal infiltration of tumor because the precise localization of the tumor infiltrated along the bile duct is crucial to present the natural history of the disease and predict survival, while it has not been included in the TNM staging system. In a study of 42 patients with cholangiocarcinoma, Zervos reported that the T subgroups were not associated with survival after resection [9]. Blumgart and Jarnagin proposed a preoperative staging system including three risk factors: biliary duct involvement, vascular involvement, and lobe atrophy [8,19]. It was the first attempt to predict resectability of HCCA preoperatively and showed a convincing accuracy for preoperative staging of HCCA. In a study of 225 HCCA patients [19], resectability was nearly 60% in J-B T1 tumors, 31% in J-B T2 tumors, and 0% of J-B T3 tumors. J-B classification has been proved to be valuable for predicting resectability and survival, while it is still inadequate for full assessment of the tumor extent and does not exceed TNM staging system on accuracy of predicting survival. In a recent study of MSKCC, the median survival of J-B T1 and J-B T2 were 22.8 and 23.0 months, respectively, and there was no significant difference [11]. Our study showed the median survivals were 27, 21, and 9 months for J-B T1, T2, and T3 stages, respectively, similar to that of MSKCC. It seems difficult to differentiate the survival between the groups of J-B T1 and T2 stages.

So, it is still needed to better stratify the patients with potential resectable HCCA, guiding surgical treatment and predicting survival. For the purpose of setting up a practical staging system, we analyzed the correlations between clinicopathological variables and survival. The results showed that J-B classification, N2 LN involvement, distant metastasis, and tumor differentiation were risk factors correlated with survival. Consistent results have been reported in the previous studies [13,15,19]. We set the scoring system and staging criteria according to the following reasons: 1. The total score of 2 to 6 was considered as stage III, which exists in the following conditions: J-B T3 stage or TNM IV stage or R2 resection. They are all indicators of poor survival less than 1 year. 2. The patients with J-B T3 stage is considered as stage III and recommended for nonsurgical therapy by the J-B classification strategy. 3. Poor differentiation, J-B T2 stage, and R1 resection are labels of advance stage of tumor and predict poor survival [6,20]. So, the patients with one of the following risk factors, J-B T2 stage, poor differentiation, or R1 resection, have a poor survival and they were considered as stage II in the current staging system. The stage of modified J-B classification is determined after operation, more quickly than TNM staging system. Patients and their families can be informed immediately about the prognosis of the disease as soon as the operation is completed, which is helpful to alleviate their anxiety for postoperative prognosis.

Compared with another staging system which is proposed by the International Cholangiocarcinoma Group and specially designed for the staging of HCCA [21], our staging system is more easy to use and practical for surgeons. The new staging system showed considerable accuracy for predicting survival. The group of stage I had a significant higher survival rate than that of stage II. The total accuracy for predicting survival was 69.5%, significantly higher than that of J-B classification and TNM staging system.

With the modified J-B staging system, it is still hard to predict the survival of the patients with a total score of 2, although they were classified to stage III. The actual survival of these patients in the study ranged from 6 to 20 months, and 60% of them survived longer than 12 months. It is possible that these patients can obtain a longer survival by an aggressive treatment.

## Conclusions

As a preoperative classification method, Jarnagin-Blumgart classification was more useful than B-C classification, while its value for predicting survival did not exceed TNM staging system. The new staging system, based on J-B classification, provides a better method to stratify HCCA patients during the operation. In the absence of effective adjuvant therapies for HCCA, the new intraoperative staging system will contribute to predict survival and guide surgical treatment.

## Abbreviations

B-C: Bismuth-Corlette
HCCA: hilar cholangiocarcinoma
J-B: Jarnagin-Blumgart
MSKCC: Memorial Sloan-Kettering Cancer Center
